# Supplementary material for: Wearable Devices for Biofeedback Rehabilitation: A Systematic Review and Meta-Analysis to Design Application Rules and Estimate the Effectiveness on Balance and Gait Outcomes in Neurological Diseases
Source: Sensors (Basel). 2021 May 15;21(10):3444. doi: 10.3390/s21103444 (PMC8156914; doi:10.3390/s21103444)
Supplement: Supplementary file 1 [file sensors-21-03444-s001.zip › sensors-1160067-supplementary.pdf]

**PUBMED**	(Humans[mh] OR Adult[mh] OR Nervous System Diseases[mh] OR Gait Disorders, Neurologic[mh] AND (Neurofeedback[mh] OR Feedback,Sensory[mh] OR feedback[tiab] OR Biofeedback[tiab] OR Cues[mh] OR Physical Therapy modalities[mh] OR Rehabilitation[mh] OR Rehab*[tiab] OR Conservative treatment[mh] OR Training[tiab] OR Exercise*[tiab]) AND (Wearable Electronic Devices[mh] OR wearable[tiab] OR Device[tiab] OR Accelerometry[mh] OR Acceleromet*[tiab] OR gyroscope*[tiab] OR sensor*[tiab] OR shoe*[tiab] OR Insole*[tiab]) AND (Walking[mh] OR Walk*[tiab] OR Ambulation[tiab] OR Gait[mh] OR Gait[tiab] OR Postural Balance[mh] OR Balance[tiab] OR Equilibrium[tiab] OR Recovery of function[mh] OR Motor Activity[mh]) AND (Randomized controlled trial[pt] OR randomized controlled trials as topic[mh] OR random*[tiab])
**COCHRANE**	(Humans OR Adult OR Nervous System Diseases OR Gait Disorders) AND (Neurofeedback OR Feedback OR Biofeedback OR Cues OR Physical Therapy OR Rehabilitation OR Conservative treatment OR Training OR Exercise) AND (Wearable Electronic Devices OR Device OR Accelerometry OR gyroscope OR sensor OR shoe OR Insole) AND (Walking OR Walk* OR Ambulation OR Gait OR Postural Balance OR Balance OR Equilibrium OR Recovery of function OR Motor Activity)
**WEB OF SCIENCE – All Databes - (MEDLINE, Web of science Core Collection) – Document Type "Clinical Trial"**	(Humans OR Adult OR Nervous System Diseases OR Gait Disorders) AND (Neurofeedback OR Feedback OR Biofeedback OR Cues OR Physical Therapy OR Rehabilitation OR Conservative treatment OR Training OR Exercise) AND (Wearable Electronic Devices OR Device OR Accelerometry OR gyroscope OR sensor OR shoe OR Insole) AND (Walking OR Walk* OR Ambulation OR Gait OR Postural Balance OR Balance OR Equilibrium OR Recovery of function OR Motor Activity)
**PEDRO:** Simple search Title, Abstract – Filtro "clinical trials"	Neurologic, Feedback, Device, Accelerometry, Gyroscope, Sensor, Balance, Gait

Table S1 – Search strategies

First Author[Ref]	WDBR intervention	Control intervention	Outcome	Level of significance between groups (p-value)
**Ginis et al.[39]**	EG: received weekly home visit and patients were instructed to walk with the CUPID system	CG: received weekly home visit by the researcher who gave advice on gait and freezing and patients were instructed to walk without using the CUPID system	2MWT	no significant differences
**Byl et al.[36]**	EG: visual kinematic feedback on the computer screen during progressive and task oriented balance and gait training activities	CG: balance and gait training activities	5TSS	no significant differences
**Byl et al.[36]**	EG: visual kinematic feedback on the computer screen during progressive and task oriented balance and gait training activities	CG: balance and gait training activities	6MWT	no significant differences
**Carpinella et al.[37]**	EG: balance and gait functional tailored exercises using Gamepad System	CG: personalized balance and gait exercises defined by the clinical staff	ABC	no significant differences
**Intiso et al.[46]**	EG: EMG Biofeedback and Physical Therapy (standard exercise bobath, facilitation and inhibition techniques, neurofacilitatory techniques)	CG: Physical therapy (standard exercise bobath, facilitation and inhibition techniques, neurofacilitatory techniques)	ankle angle (heel contact)	no significant differences
**Cozean et al.[45]**	EG1: EMG biodeedback during static and dynamic activities<br>EG2: FES during static and dynamic activities<br>EG3: EMG biofeedback+FES during static and dynamic activities	CG: conventional physical therapy	ankle angle (swing phase)	significant differences ($p = 0.02$) in favour of EG3
**Intiso et al.[46]**	EG: EMG Biofeedback and Physical Therapy (standard exercise bobath, facilitation and inhibition techniques, neurofacilitatory techniques)	CG: Physical therapy (standard exercise bobath, facilitation and inhibition techniques, neurofacilitatory techniques)	ankle angle (swing phase)	significant differences ($p < 0.02$) in favour of experimental group
**Jonsdottir et al.[47]**	EG=Task oriented Gait Training with EMG-BFB device	CG= conventional physical therapy (at least 15 minutes of gait training in each session)	ankle power peak at push-off	significant differences ($p = 0.16$) in favour of experimental group

**Intiso et al.[46]**	EG: EMG Biofeedback and Physical Therapy (standard exercise bobath, facilitation and inhibition techniques, neurofacilitatory techniques)	CG: Physical therapy (standard exercise bobath, facilitation and inhibition techniques, neurofacilitatory techniques)	Basmajian scale	significant differences ($p < 0.01$) in favour of experimental group
**Byl et al.[36]**	EG: visual kinematic feedback on the computer screen during progressive and task oriented balance and gait training activities	CG: balance and gait training activities	BBS	no significant differences
**van den Heuvel et al.[40]**	EG: interactive balance games with explicit augmented visual feedback	CG: conventional balance training recommended by the guidelins for physical therapy.	BBS	no significant differences
**Azerpaikan et al.[35]**	EG: Neurofeedback training with EEG generator	CG: Sham Neuro Feedback Training using sham EEG generator	BBS	significant differences ($p = 0.00$) in favour of experimental group
**Lee et al.[50]**	EG: neurofeedback training	CG: pseudo-neurofeedback training (sham neurofeedback)	cadence	significant differences ($p < 0.05$) in favour of experimental group
**El-Tamawy et al.[38]**	EG: Individually designed physiotherapy and traditional gait training plus tredmill training with vibratory stimuly	CG: individually designed physiotherapy traditional gait training including instructions to walk with long steps.	cadence	significant differences ($p = 0.001$) in favour of experimental group
**Schwenk et al.[54]**	EG: postural balance exercises during standing using biofeedback training	CG: No training;	CoM area EC	no significant differences
**Schwenk et al.[54]**	EG: postural balance exercises during standing using biofeedback training	CG: No training;	CoM area EO	significant differences ($p = 0.04$) in favour of experimental group
**Schwenk et al.[54]**	EG: postural balance exercises during standing using biofeedback training	CG: No training;	CoM sway AP-EC	no significant differences
**Schwenk et al.[54]**	EG: postural balance exercises during standing using biofeedback training	CG: No training;	CoM sway AP-EO	significant differences ($p = 0.02$) in favour of experimental group
**Schwenk et al.[54]**	EG: postural balance exercises during standing using biofeedback training	CG: No training;	CoM sway ML-EC	no significant differences
**Schwenk et al.[54]**	EG: postural balance exercises during standing using biofeedback training	CG: No training;	CoM sway ML-EO	significant differences ($p = 0.04$) in favour of experimental group
**Carpinella et al.[37]**	EG: balance and gait functional tailored exercises using Gamepad System	CG: personalized balance and gait exercises defined by the clinical staff	COP AP sway	no significant differences

**Carpinella et al.**[37]	EG: balance and gait functional tailored exercises using Gamepad System	CG: personalized balance and gait exercises defined by the clinical staff	COP ML sway	significant differences ($p = 0.003$) in favour of experimental group
**Choi et al.**[44]	EG: gait intervention with auditory feedback	CG= general gait intervention	COP lenght EC	significant differences ($p < 0.05$) in favour of experimental group
**Cha et al.**[42]	EG1: gait training with active weight bearing on the paretic heel with auditory feedback EG2: gait training with auditory feedback from paretic metatarsals	CG: gait intervention	COP lenght EC	no significant differences
**Choi et al.**[44]	EG: gait intervention with auditory feedback	CG= general gait intervention	COP lenght EO	significant differences ($p < 0.05$) in favour of experimental group
**Cha et al.**[42]	EG1: gait training with active weight bearing on the paretic heel with auditory feedback EG2: gait training with auditory feedback from paretic metatarsals	CG: gait intervention	COP lenght EO	significant differences ($p < 0.017$) in favour of experimental groups compared to control
**Lupo et al.**[51]	EG: balance training with RIABLO biofeedback system using a video interface.	CG: conventional balance training without the use of the RIABLO biofeedback system	COP length EC	significant differences ($p = 0.0002$) in favour of experimental group
**Lupo et al.**[51]	EG: balance training with RIABLO biofeedback system using a video interface.	CG: conventional balance training without the use of the RIABLO biofeedback system	COP length EO	significant differences ($p < 0.0001$) in favour of experimental group
**Cha et al.**[42]	EG1: gait training with active weight bearing on the paretic heel with auditory feedback EG2: gait training with auditory feedback from paretic metatarsals	CG: gait intervention	COP velocity EC	no significant differences
**Cha et al.**[42]	EG1: gait training with active weight bearing on the paretic heel with auditory feedback EG2: gait training with auditory feedback from paretic metatarsals	CG: gait intervention	COP velocity EO	significant differences ($p < 0.017$) in favour of experimental groups compared to control
**Byl et al.**[36]	EG: visual kinematic feedback on the computer screen during progressive and task oriented balance and gait training activities	CG: balance and gait training activities	DGI	no significant differences

**Ginis et al.**[39]	EG: received weekly home visit and patients were instructed to walk with the CUPID system	CG: received weekly home visit by the researcher who gave advice on gait and freezing and patients were instructed to walk without using the CUPID system	double support time	no significant differences
**Ginis et al.**[39]	EG: received weekly home visit and patients were instructed to walk with the CUPID system	CG: received weekly home visit by the researcher who gave advice on gait and freezing and patients were instructed to walk without using the CUPID system	double support time - DT	no significant differences
**Ginis et al.**[39]	EG: received weekly home visit and patients were instructed to walk with the CUPID system	CG: received weekly home visit by the researcher who gave advice on gait and freezing and patients were instructed to walk without using the CUPID system	FES-I	no significant differences
**Schwenk et al.**[54]	EG: postural balance exercises during standing using biofeedback training	CG: No training;	FES-I	significant differences (p = 0.02) in favour of experimental group
**van den Heuvel et al.**[40]	EG: interactive balance games with explicit augmented visual feedback	CG: conventional balance training recommended by the guidelins for physical therapy.	FES-I	no significant differences
**Cha et al.**[42]	EG1: gait training with active weight bearing on the paretic heel with auditory feedback EG2: gait training with auditory feedback from paretic metatarsals	CG: gait intervention	FGA	significant differences (p < 0.017) in favour of EG2 compared to control
**Choi et al.**[44]	EG: gait intervention with auditory feedback	CG= general gait intervention	FGA	significant differences (p < 0.05) in favour of experimental group
**Byl et al.**[36]	EG: visual kinematic feedback on the computer screen during progressive and task oriented balance and gait training activities	CG: balance and gait training activities	FOG-Q	no significant differences
**Carpinella et al.**[37]	EG: balance and gait functional tailored exercises using Gamepad System	CG: personalized balance and gait exercises defined by the clinical staff	FOG-Q	no significant differences
**Ginis et al.**[39]	EG: received weekly home visit and patients were instructed to walk with the CUPID system	CG: received weekly home visit by the researcher who gave advice on gait and freezing and patients were instructed to walk without using the CUPID system	FSST	no significant differences

**Byl et al.[36]**	EG: visual kinematic feedback on the computer screen during progressive and task oriented balance and gait training activities	CG: balance and gait training activities	gait speed	no significant differences
**Cho et al.[43]**	EG: visual biofeedback tracking training	CG: not Reported	gait speed	no significant differences
**Cozean et al.[45]**	EG1: EMG biodeedback during static and dynamic activities<br>EG2: FES during static and dynamic activities<br>EG3: EMG biofeedback+FES during static and dynamic activities	CG: conventional physical therapy	gait speed	significant differences (p = 0.04) in favour of EG3
**El-Tamawy et al.[38]**	EG: Individually designed physiotherapy and traditional gait training plus tredmill training with vibratory stimuly	CG: individually designed physiotherapy traditional gait training including instructions to walk with long steps.	gait speed	significant differences (p = 0.001) in favour of experimental group
**Jonsdottir et al.[47]**	EG=Task oriented Gait Training with EMG-BFB device	CG= conventional physical therapy (at least 15 minutes of gait training in each session)	gait speed	significant differences (p = 0.004) in favour of experimental group
**Lee et al.[50]**	EG: neurofeedback training	CG: pseudo-neurofeedback training (sham neurofeedback)	gait speed	significant differences (p < 0.05) in favour of experimental group
**Mandel et al.[52]**	EG1:EMG biofeedback training<br>EG2: EMG biofeedback followed by rhythmic positional biofeedback	CG: nothing	gait speed	significant differences (p = 0.02) in favour of EG2
**van den Heuvel et al.[40]**	EG: interactive balance games with explicit augmented visual feedback	CG: conventional balance training recommended by the guidelins for physical therapy.	gait speed	no significant differences
**Intiso et al.[46]**	EG: EMG Biofeedback and Physical Therapy (standard exercise bobath, facilitation and inhibition techniques, neurofacilitatory techniques)	CG: Physical therapy (standard exercise bobath, facilitation and inhibition techniques, neurofacilitatory techniques)	gait speed	no significant differences
**Ginis et al.[39]**	EG: received weekly home visit and patients were instructed to walk with the CUPID system	CG: received weekly home visit by the researcher who gave advice on gait and freezing and patients were instructed to walk without using the CUPID system	gait speed - DT	no significant differences

**Schwenk et al.[54]**	EG: postural balance exercises during standing using biofeedback training	CG: No training	gait speed-fast	no significant differences
**Schwenk et al.[54]**	EG: postural balance exercises during standing using biofeedback training	CG: No training	gait speed-normal	no significant differences
**Schwenk et al.[54]**	EG: postural balance exercises during standing using biofeedback training	CG: No training	gait stride time variability-fast	no significant differences
**Schwenk et al.[54]**	EG: postural balance exercises during standing using biofeedback training	CG: No training	gait stride time variability-normal	no significant differences
**Cozean et al.[45]**	EG1: EMG biodeedback during static and dynamic activities<br>EG2: FES during static and dynamic activities<br>EG3: EMG biofeedback+FES during static and dynamic activities	CG: conventional physical therapy	knee angle (swing phase)	significant differences (p = 0.05) in favour of EG3
**Jonsdottir et al.[47]**	EG=Task oriented Gait Training with EMG-BFB device	CG= conventional physical therapy (at least 15 minutes of gait training in each session)	knee flexion peak	no significant differences
**Azerpaikan et al.[35]**	EG: Neurofeedback training with EEG generator	CG: Sham Neuro Feedback Training using sham EEG generator	limit of stability	significant differences (p = 0.00) in favour of experimental group
**Sungkarat, 2011**	EG: conventional rehabilitation and gait training with I-ShoWS set-up	CG: conventional rehabilitation and gait training without I-ShoWS set-up	load on paretic leg during stance (% body weight)	significant differences (p = 0.004) in favour of experimental group
**Ginis et al.[39]**	EG: received weekly home visit and patients were instructed to walk with the CUPID system	CG: received weekly home visit by the researcher who gave advice on gait and freezing and patients were instructed to walk without using the CUPID system	MinibestTEST	significant differences (p = 0.04) in favour of experimental group
**Cho et al.[43]**	EG: visual biofeedback tracking training	CG: not Reported	Modified Motor Assessment Scale (walking item only)	no significant differences
**Cho et al.[43]**	EG: visual biofeedback tracking training	CG: not Reported	Motoricity Index	no significant differences
**Ginis et al.[39]**	EG: received weekly home visit and patients were instructed to walk with the CUPID system	CG: received weekly home visit by the researcher who gave advice on gait and freezing and patients were instructed to walk without using the CUPID system	NFOG-Q	no significant differences

**Ginis et al.**[39]	EG: received weekly home visit and patients were instructed to walk with the CUPID system	CG: received weekly home visit by the researcher who gave advice on gait and freezing and patients were instructed to walk without using the CUPID system	PASE	no significant differences
**Jung et al.**[48]	EG: gait training with auditory feedback	CG: gait training without auditory feedback	peak force cane	significant differences (p = 0.01) in favour of experimental group
**Lee et al.**[50]	EG: neurofeedback training	CG: pseudo-neurofeedback training (sham neurofeedback)	plantar foot pressure (entire foot-during dual task activitiy)	significant differences (p < 0.01) in favour of experimental group
**Lee et al.**[50]	EG: neurofeedback training	CG: pseudo-neurofeedback training (sham neurofeedback)	plantar foot pressure (forefoot- during dual task activitiy)	significant differences (p < 0.05) in favour of experimental group
**Lee et al.**[50]	EG: neurofeedback training	CG: pseudo-neurofeedback training (sham neurofeedback)	plantar foot pressure (hindfoot-during dual task activitiy)	no significant differences
**Lupo et al.**[51]	EG: balance training with RIABLO biofeedback system using a video interface.	CG: conventional balance training without the use of the RIABLO biofeedback system	RMI	no significant differences
**Mandel et al.**[52]	EG1:EMG biofeedback training<br>EG2: EMG biofeedback followed by rhythmic positional biofeedback	CG: nothing	ROM (Plantar Flexion)	significant differences (p = 0.01) in favour of EG1
**Jung et al.**[48]	EG: gait training with auditory feedback	CG: gait training without auditory feedback	single leg stance (affected side)	significant differences (p = 0.03) in favour of experimental group
**van den Heuvel et al.**[40]	EG: interactive balance games with explicit augmented visual feedback	CG: conventional balance training recommended by the guidelins for physical therapy.	single leg stance test	no significant differences
**Ki et al.**[49]	EG: neuro-developmental treatment with auditory feedback	CG: neuro-developmental treatment	single limb stance (affected side)	no significant differences
**Jung et al.**[48]	EG: gait training with auditory feedback	CG: gait training without auditory feedback	single support time	significant differences (p = 0.02) in favour of experimental group
**Sungkarat, 2011**	EG: conventional rehabilitation and gait training with I-ShoWS set-up	CG: conventional rehabilitation and gait training without I-ShoWS set-up	single support time asimmetry ratio	significant differences (p = 0.03) in favour of experimental group

**Ki et al.[49]**	EG: neuro-developmental treatment with auditory feedback	CG: neuro-developmental treatment	stance phase duration	no significant differences
**Lee et al.[50]**	EG: neurofeedback training	CG: pseudo-neurofeedback training (sham neurofeedback)	stance phase percentage (during dual task activitiy)	no significant differences
**Byl et al.[36]**	EG: visual kinematic feedback on the computer screen during progressive and task oriented balance and gait training activities	CG: balance and gait training activities	step length	no significant differences
**Intiso et al.[46]**	EG: EMG Biofeedback and Physical Therapy (standard exercise bobath, facilitation and inhibition techniques, neurofacilitatory techniques)	CG: Physical therapy (standard exercise bobath, facilitation and inhibition techniques, neurofacilitatory techniques)	step length	no significant differences
**Sungkarat, 2011**	EG: conventional rehabilitation and gait training with I-ShoWS set-up	CG: conventional rehabilitation and gait training without I-ShoWS set-up	step length asimmetry ratio	significant differences (p = 0.03) in favour of experimental group
**Aruin et al.[41]**	EG: Conventional balance and gait therapy combined with auditory biofeedback	CG: Conventional balance and gait therapy during which patients occasionally received verbal information about BOS	step width	significant differences (p<0.05) in favour of experimental group
**El-Tamawy et al.[38]**	EG: Individually designed physiotherapy and traditional gait training plus tredmill training with vibratory stimuly	CG: individually designed physiotherapy traditional gait training including instructions to walk with long steps.	stride length	significant differences (p = 0.001) in favour of experimental group
**Ginis et al.[39]**	EG: received weekly home visit and patients were instructed to walk with the CUPID system	CG: received weekly home visit by the researcher who gave advice on gait and freezing and patients were instructed to walk without using the CUPID system	stride length	no significant differences
**Jonsdottir et al.[47]**	EG=Task oriented Gait Training with EMG-BFB device	CG= conventional physical therapy (at least 15 minutes of gait training in each session)	stride length	significant differences (p = 0.001) in favour of experimental group
**Ginis et al.[39]**	EG: received weekly home visit and patients were instructed to walk with the CUPID system	CG: received weekly home visit by the researcher who gave advice on gait and freezing and patients were instructed to walk without using the CUPID system	stride length - DT	no significant differences

**Byl et al.**[36]	EG: visual kinematic feedback on the computer screen during progressive and task oriented balance and gait training activities	CG: balance and gait training activities	TBS	no significant differences
**Byl et al.**[36]	EG: visual kinematic feedback on the computer screen during progressive and task oriented balance and gait training activities	CG: balance and gait training activities	TUG	no significant differences
**Ki et al.**[49]	EG: neuro-developmental treatment with auditory feedback	CG: neuro-developmental treatment	TUG	significant differences ($p < 0.01$) in favour of experimental group
**Carpinella et al.**[37]	EG: balance and gait functional tailored exercises using Gamepad System	CG: personalized balance and gait exercises defined by the clinical staff	UPDRSIII	no significant differences
**Ginis et al.**[39]	EG: received weekly home visit and patients were instructed to walk with the CUPID system	CG: received weekly home visit by the researcher who gave advice on gait and freezing and patients were instructed to walk without using the CUPID system	UPDRSIII	no significant differences
**van den Heuvel et al.**[40]	EG: interactive balance games with explicit augmented visual feedback	CG: conventional balance training recommended by the guidelins for physical therapy.	UPDRSIII	significant differences ($p = 0.02$) in favour of experimental group
**El-Tamawy et al.**[38]	EG: Individually designed physiotherapy and traditional gait training plus tredmill training with vibratory stimuly	CG: individually designed physiotherapy traditional gait training including instructions to walk with long steps.	walking distance (treadmill)	significant differences ($p = 0.001$) in favour of experimental group

Table S2 – Qualitative Synthesis
